# Dietary Provision, GLIM-Defined Malnutrition and Their Association with Clinical Outcome: Results from the First Decade of nutritionDay in China

**DOI:** 10.3390/nu16040569

**Published:** 2024-02-19

**Authors:** Bei Zhou, Yupeng Zhang, Michael Hiesmayr, Xuejin Gao, Yingchun Huang, Sitong Liu, Ruting Shen, Yang Zhao, Yao Cui, Li Zhang, Xinying Wang, on behalf of the nutritionDay Chinese Working Group

**Affiliations:** 1 Department of General Surgery, Jinling Hospital, Medical School of Nanjing University, 305 East Zhongshan Road, Nanjing 210002, China; zhoubei_198@njucm.edu.cn (B.Z.); zhangyupeng@smail.nju.edu.cn (Y.Z.); 522023350251@smail.nju.edu.cn (X.G.);; 2 Department of Nutrition, Acupuncture, Moxibustion and Massage College, Health Preservation and Rehabilitation College, Nanjing University of Chinese Medicine, 138 Xianlin Road, Nanjing 210023, China; 3 Center for Medical Data Science, Section for Medical Statistics, Medical University Vienna, Spitalgasse 23, A-1090 Vienna, Austria; michael.hiesmayr@meduniwien.ac.at; 4 Department of Biostatistics, School of Public Health, Nanjing Medical University, 101 Longmian Avenue, Nanjing 211166, China; yzhao@njmu.edu.cn; 5 Department of Nutrition, Pizhou Hospital, Xuzhou Medical University, Xuzhou 221004, China; 760020240003@xzhmu.edu.cn

**Keywords:** dietary provision, GLIM criteria, hospitalized patients, nutritionDay, nutritional status

## Abstract

Malnutrition is a common and serious issue that worsens patient outcomes. The effects of dietary provision on the clinical outcomes of patients of different nutritional status needs to be verified. This study aimed to identify dietary provision in patients with eaten quantities of meal consumption and investigate the effects of dietary provision and different nutritional statuses defined by the GLIM criteria on clinical outcomes based on data from the nutritionDay surveys in China. A total of 5821 adult in-patients from 2010 to 2020 were included in this study’s descriptive and Cox regression analyses. Rehabilitation and home discharge of 30-day outcomes were considered a good outcome. The prevalence of malnutrition defined by the GLIM criteria was 22.8%. On nutritionDay, 51.8% of all patients received dietary provisions, including hospital food and a special diet. In multivariable models adjusting for other variables, the patients receiving dietary provision had a nearly 1.5 higher chance of a good 30-day outcome than those who did not. Malnourished patients receiving dietary provision had a 1.58 (95% CI [1.36–1.83], *p* < 0.001) higher chance of having a good 30-day outcome and had a shortened length of hospital stay after nutritionDay (median: 7 days, 95% CI [6–8]) compared to those not receiving dietary provision (median: 11 days, 95% CI [10–13]). These results highlight the potential impacts of the dietary provision and nutritional status of in-patients on follow-up outcomes and provide knowledge on implementing targeted nutrition care.

## 1. Introduction

Hospital malnutrition has been gaining attention due to its increased incidence. Malnutrition may be related to in-patients’ disease stage, economic situation, or other health problems [1,2,3]. China has participated in the nutritionDay initiative to fight malnutrition in hospital settings since 2010 [4]. nutritionDay is an annual single-day, multinational, cross-sectional audit with 30-day follow-up outcomes [5,6]. With the inspirational global call to action and increasing awareness of malnutrition, the voluntary participation of hospitals in nutritionDay has been expanding. The Chinese nutritionDay Working Group has been established for 10 years and concentrates on real-world studies of in-patients’ nutritional status and nutrition intake.

The early identification of malnutrition, followed by timely and appropriate intervention, can significantly improve clinical outcomes and benefit in-patients [1,7,8]. To promote the global use of standardized diagnostic criteria, the Global Leadership Initiative on Malnutrition (GLIM) developed a two-step approach of risk screening and diagnostic assessment to identify malnutrition [9]. The GLIM considered reduced food intake as one of the etiologic criteria for malnutrition. In addition, nutrition intake, including dietary provision and artificial nutrition, is more complicated during nutrition management.

On the one hand, dietary provision is the basis of medical interventions focusing on patients’ daily lives [10]. As reported in previous studies, dietary sources of omega-3 fatty acids were recommended instead of supplements in patients with ulcerative colitis [11]; plant-based diets were associated with decreased risk of metabolic syndrome [12,13]; a higher frequency of maternal Mediterranean-style diet was associated with a lower likelihood of neurodevelopmental disabilities in offspring [14]. On the other hand, artificial nutrition should be properly provided to supplement daily metabolic nutrition requirements, particularly in malnourished patients with inadequate dietary intakes [15,16]. However, previous studies on China nutritionDay surveys in single years have highlighted an inappropriate level of nutritional therapy and indicated that patients who needed artificial nutrition were associated with a prolonged length of hospital stay (LOS) [4,17]. Therefore, more attention should be paid to whether adequate food or diet is available in daily nutritional care, especially in malnourished patients. Although the meal consumption of in-patients on nutritionDay was revealed to be negatively associated with mortality [18], evidence on the effect of dietary provision with meal intake is limited [5,18]. Moreover, the effects of dietary provision and different nutritional status on clinical outcomes remain unknown.

Therefore, based on data from the nutritionDay surveys in China, the present study aimed to (1) identify dietary provision in patients with different quantities of meal consumption and (2) investigate the effects of dietary provision and different nutritional statuses defined by the GLIM criteria on clinical outcomes.

## 2. Materials and Methods

### 2.1. Study Population

**Figure 1:**
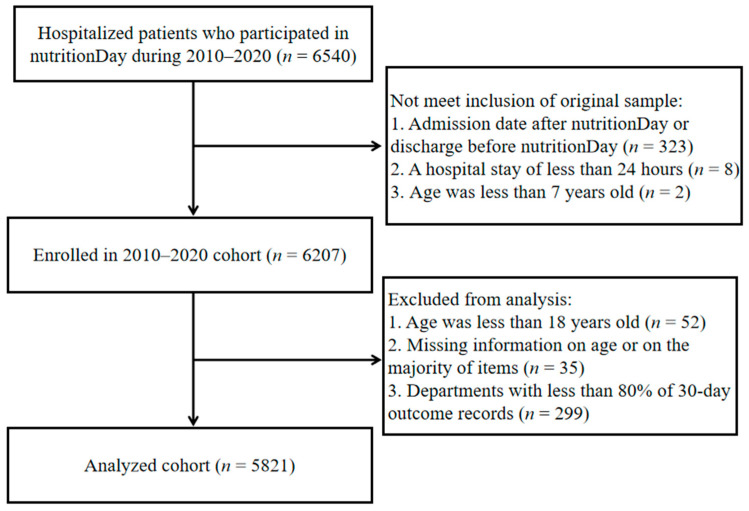
Flowchart describing the selection of study subjects (2010–2020 nutritionDay in China).

By the end of 2020, 20 Chinese tertiary referral hospitals had participated in the multicenter nutritionDay study. In-hospital patients (excluding patients in the ICU) were prospectively registered for the survey day every November. Patients were excluded if they were under 18 years of age or had missing information on age or the majority of items; participating departments with less than 80% of 30-day outcome records were also excluded (Figure 1). The nutritionDay project was conducted in accordance with the Declaration of Helsinki and approved by the Institutional Review Board (or Ethics Committee) of the Medical University of Vienna (EK407/2005). In accordance with national regulations, this study was also approved by the Ethics Committee of the Jinling Hospital (a Chinese host hospital) and amended annually (approval code 2022DZKY-067-01; date of approval 22 June 2022). All patients provided signed informed consent and were informed of their right to refuse to participate before the survey.

### 2.2. Data Collection

Patient nutritional status and clinical outcomes from 2010 to 2020 were obtained from the nutritionDay dataset of China. Data were collected separately through standardized questionnaires from the nutritionDay website https://www.nutritionday.org/en/-35-.languages/languages.html (accessed on 2 February 2024) for each survey year, categorized into three parts. Part 1 reflected the general situation of the hospital and unit. Part 2 described the patients’ characteristics, the clinical information, and the outcome recorded by clinical staff. Part 3 consisted of medical history, nutrition intake, and health status from the patient’s perspective. Patients reported their mobility, weight change, food intake history, and meal consumption on nutritionDay, while hospital staff (including caregivers and health care professionals) reported on patient demographics, nutritional provision, and 30-day clinical outcomes. In the present study, nutritional provision was classified as dietary provision (regular hospital food, fortified/enriched hospital food, and special diet), non-dietary provision (protein/energy supplement, enteral nutrition, and parenteral nutrition), as well as multi-form of food and artificial nutrition.

### 2.3. Nutritional Status Evaluation

Nutritional risk and malnutrition were evaluated through questions regarding weight loss, disease condition, and dietary intake in the nutritionDay questionnaires. Risk screening was assessed using the Malnutrition Universal Screening Tool (MUST) [19], which has been extended to hospital settings because of its validity, as supported by previous studies [20,21,22]. The MUST score (Supplementary Table S1a) was calculated based on the patient’s BMI, weight loss, and acute disease effects. A total MUST score of ≥1 is defined as nutritionally at-risk.

Nutritional status was evaluated according to the GLIM criteria [9] for malnutrition. The GLIM system relies on the presence of nutritional risk as the basis for diagnosing malnutrition, including the presence of at least one phenotype (unintentional weight loss, low BMI, or reduced muscle mass) and one etiologic criterion (reduced food intake/assimilation or disease burden/inflammatory condition). In this study, the phenotypic criteria were derived from the patient’s weight loss and BMI, and the etiologic criteria were assessed from information about food intake in the week before admission or on the survey day and diagnosis at admission or presence of chronic disease-related comorbidities (Supplementary Table S1b) [23].

### 2.4. Outcomes

The 30-day outcomes were dichotomized as good or poor according to the outcome codes in the nutritionDay survey. Rehabilitation and home discharge were considered a good outcome, and the remaining outcomes, including still in the hospital, transfer to another hospital, transfer to long-term care, and death, were considered poor outcomes. The LOS before and after the nutritionDay were calculated for each patient. The clinical outcome parameters in this study mainly focused on the good 30-day outcome.

### 2.5. Statistical Analysis

Patient characteristics were analyzed using descriptive statistics. Continuous variables not normally distributed were expressed as a median with interquartile range (IQR), while categorical variables were expressed as counts and percentages. We used the Wilcoxon rank sum test, Chi-square test, or Fisher’s exact tests to compare differences between different groups divided by clinical outcomes or nutritional status where appropriate (Supplementary Tables S2 and S4). Among these, significant variables with *p* < 0.05 and the variables of sex and surgical status [18] were further analyzed to evaluate their association with good 30-day outcomes using Cox regression analysis individually. If significant (*p* < 0.05), these variables were included in three multivariable Cox regression models of dietary provision and malnutrition diagnosis associated with a good 30-day outcome. Model Ⅰ was used to identify the association between dietary provision and good outcomes, adjusted for departments, survey year, hospital location, sex, BMI, weight change within the last 3 months, major lesion types, comorbidity, food intake in the previous week, eating on nutritionDay, previous ICU stay, mobility, self-rated health, surgical status, LOS before nutritionDay, and number of drugs before admission. Model Ⅱ was used to identify the association between GLIM-defined malnutrition and good outcome, adjusted for departments, survey year, hospital location, sex, previous ICU stay, mobility, self-rated health, surgical status, LOS before nutritionDay, dietary provision, and the number of drugs before admission. Model Ⅲ was used to identify the association between malnutrition diagnosis and dietary provision and good outcomes, adjusted for departments, survey year, hospital location, sex, previous ICU stay, mobility, self-rated health, surgical status, LOS before nutritionDay, and the number of drugs before admission. Cumulative incidence curves of good 30-day outcomes were plotted for dietary provision and different nutritional status categories defined by the GLIM criteria. Log-rank tests were used to compare the differences between groups. These results were expressed as median or hazard ratios (HR) with their 95% confidence intervals (CI). Statistical analyses were performed using R version 4.2.1. Statistical significance was set at a *p*-value < 0.05.

## 3. Results

### 3.1. Demographic Characteristics of the Hospitalized Patients

As presented in Table 1, a total of 5821 in-patients from 20 hospitals within various departments were analyzed in this study. Of the total subjects, 40.4% were female, the median age was 58 years (IQR 45–67), and the median BMI was 22.8 kg/m^2^ (IQR 20.2–25.2). Approximately 30.9% of all patients were surgical patients. Weight loss in the previous three months was reported by 2246 patients (38.6%). Approximately 19.8% of all patients reported less than half of normal food intake in the previous week. On nutritionDays, dietary provision (in the form of food or diet) was given to 3015 in-patients (51.8%), the majority of whom received hospital food (*n* = 2699, 46.4%). Screening identified 33.1% of patients who are nutritionally at-risk according to the MUST (MUST score ≥ 1, *n* = 1924), and malnutrition based on the GLIM criteria was diagnosed in 1328 patients (22.8%). The median LOS after nutritionDay was 6 days (IQR 3.0–12.0). A good 30-day outcome, including rehabilitation and home discharge, was recorded in 5093 (87.5%) patients.

### 3.2. Dietary Provision with Meal Consumption on nutritionDay

Notably, the percentage of patients receiving dietary provision and who consumed a meal on nutritionDay were not equivalent: although 51.8% of all in-patients received dietary provision, only 37.3% of patients finished their meals. The main reason for patients eating less or nothing was that they were not allowed to eat (*n* = 968, 16.6%), followed by decreased appetite (*n* = 930, 16.0%) (Table 1). Dietary provision, including hospital food (46.4%), special diet (4.1%), and a combination of the two (1.3%), was the main source of nutritional provision for patients, whereas patients without dietary provision mainly received artificial nutrition (23.2%) and nothing else (3.3%). In patients who self-reported eating a full meal, 71.0% received dietary provisions. However, in patients eating nothing but who were allowed to eat, nearly 30% received no dietary provision, which increased to 70.3% in patients eating nothing due to not being allowed to eat (Table 2).

### 3.3. GLIM Diagnostic Flow Chart with Dietary Provision and Good 30-Day Outcome

**Figure 2:**
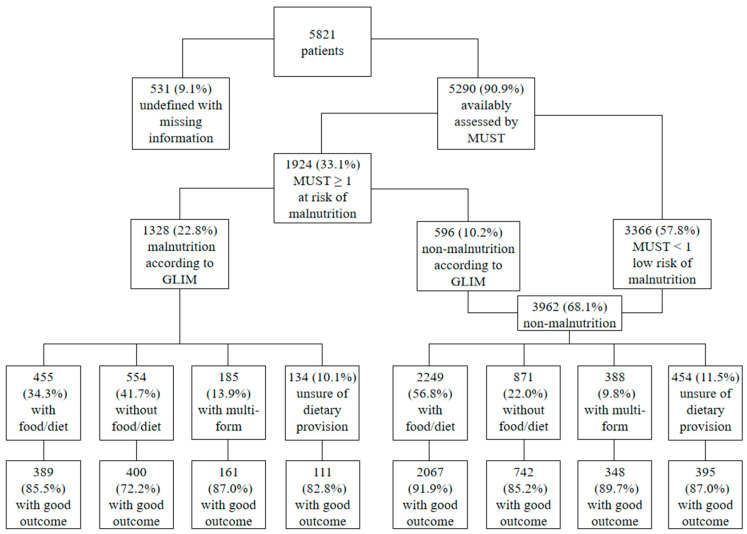
GLIM diagnostic flowchart with dietary provision and good 30-day outcome. MUST, Malnutrition Universal Screening Tool; GLIM, Global Leadership Initiative on Malnutrition.

A diagnostic flowchart of the GLIM criteria regarding dietary provision and good 30-day outcomes is presented in Figure 2. In terms of dietary provision, more than half of the non-malnourished patients received food/diet, whereas 41.7% of the malnourished patients did not receive dietary provisions. Furthermore, of the patients with GLIM-defined malnutrition (*n* = 1328, 22.8%), 85.5% of the 455 patients with dietary provision had a good outcome, whereas 72.2% of the 554 patients without dietary provision had a good outcome. In contrast, in the 2249 non-malnourished patients with dietary provision, the frequency of a good outcome increased to 91.9%.

### 3.4. Dietary Provision and Malnutrition Diagnosis Associated with Good 30-Day Outcome

Cox regression models were used to determine the association of dietary provision and malnutrition diagnosis with a good 30-day outcome (Table 3). In the univariate analysis shown in Supplementary Table S3, the HR for a good 30-day outcome was 1.55 (95% CI [1.45–1.66], *p* < 0.001) for patients with dietary provision, compared with patients not receiving food/diet. Similar trends between patients with dietary provision and a good 30-day outcome were also found in the multivariable analyses of model Ⅰ (HR 1.47, 95% CI [1.35–1.60], *p* < 0.001) and model Ⅱ (HR 1.49, 95% CI [1.38–1.61], *p* < 0.001). Model Ⅱ revealed a negative relationship between malnutrition defined by the GLIM criteria (HR 0.83, 95% CI [0.77–0.89], *p* < 0.001) and a good 30-day outcome. However, when malnutrition combined with dietary provision was included in model Ⅲ, it was found that compared with malnourished patients without dietary provision, malnourished patients receiving food/diet (HR 1.58, 95% CI [1.36–1.83], *p* < 0.001), malnourished patients receiving multi-form of food and artificial nutrition (HR 1.34, 95% CI [1.11–1.63], *p* = 0.003), and non-malnourished patients receiving food/diet (HR 1.86, 95% CI [1.64–2.11], *p* < 0.001) were significantly associated with increased good 30-day outcomes.

### 3.5. Cumulative Incidence of Good Outcome within 30 Days after nutritionDay

**Figure 3:**
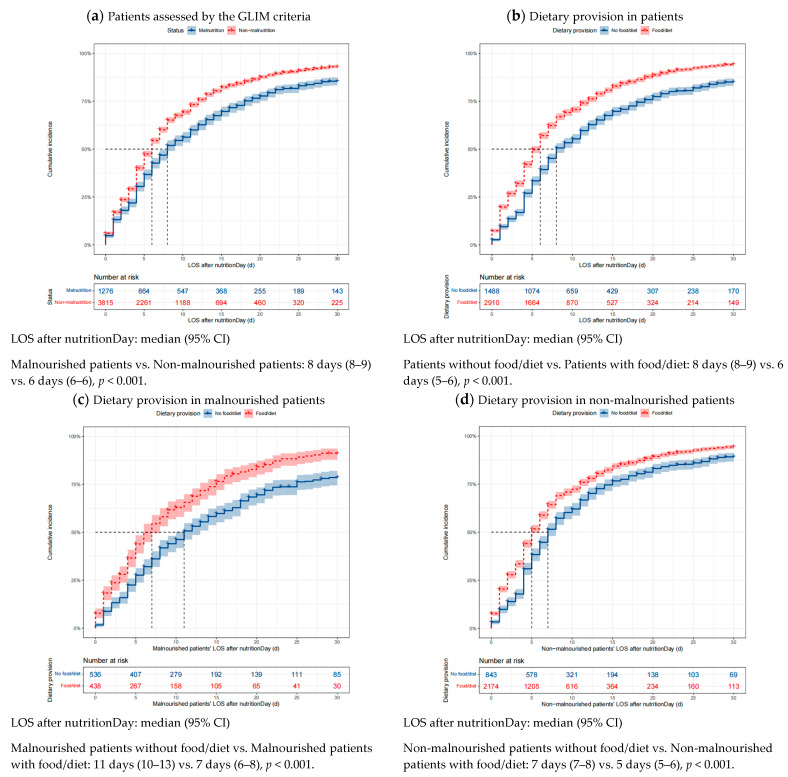
Cumulative incidence of good outcomes within 30 days after nutritionDay in patients with different nutritional status and dietary provision. Missing data were excluded. Differences in the median LOS after nutritionDay between groups were tested using the log-rank test. Shaded areas indicate 95% CI. LOS, length of stay in hospital; GLIM, Global Leadership Initiative on Malnutrition; CI, confidence interval.

The good 30-day outcomes in patients with different nutritional status and dietary provisions are visualized using cumulative incidence curves in Figure 3. Patients with malnutrition defined using the GLIM criteria had a median LOS of 8 days after nutritionDay, whereas non-malnourished patients had a median LOS of 6 days after nutritionDay (*p* < 0.001). Similar correlations can be observed for the association between dietary provision and good 30-day outcomes. Moreover, malnourished patients provided with food/diet had a significantly shortened median LOS after nutritionDay compared with those not receiving food/diet (7 days vs. 11 days, *p* < 0.001). Likewise, non-malnourished patients receiving food/diet also had a significantly shortened median LOS after nutritionDay compared with those not receiving dietary provision (5 days vs. 7 days, *p* < 0.001).

## 4. Discussion

To our knowledge, this is the first study to focus on the 30-day outcomes of in-patients in association with dietary provision and nutritional status as defined by the GLIM criteria. The results showed that more than half of the patients participating in the Chinese 2010–2020 nutritionDay cohort received dietary provision, especially in patients who reported full meal consumption on nutritionDay. In the multivariable models adjusted for other variables, dietary provision was associated with increased good 30-day outcomes compared to non-dietary provision, even in malnourished patients.

### 4.1. Dietary Provision with Meal Consumption on nutritionDay

In total, 62.3% of the patients in the Chinese cohort received dietary provisions with any oral diet on nutritionDay compared with 80.9% and 74% in the analyses of Polish results [24] and European data [5], respectively. Moreover, 58% of the patients in Chinese hospitals did not finish their meals, compared with 55% in European hospitals [24]. However, 16.6% of patients who reported eating less in this sample were not allowed to eat, in contrast to 5% of such patients in the European data [24].

The more patients that are allowed to eat and the more dietary provisions administered by hospital staff, the greater number of patients who might at least have some food intake. Among the patients who consumed their full meal on nutritionDay, about 71.0% received dietary provision from hospital food and a special diet, similar to the rate of 75.7% reported in the U.S. [18], but lower than that reported in European hospitals at 84% [5]. The higher rate of dietary provision in Europe might be associated with sustainable nutrition policies and practices [25,26,27].

Sustainable diets in nutrition policies are reflective of orientation and focus, engagement styles, and modes of leadership [25]. Dietary provision during clinical nutrition management requires careful collaboration across departments and good governance of evidence [28] regarding comparable surveillance data on key indicators and their determinants [26,27]. Evidence-based nutrition policy and approaches to evidence-based practice require the cooperation of nutrition researchers, policymakers, and practitioners to build a flexible scientific framework for dietary provision and monitor dietary intake systems [29].

A cross-sectional study of dietary intakes conducted by Bannerman indicated the need for greater monitoring of patient food consumption [30]. As meal consumption on nutritionDay is considered one of the etiologic criteria in the GLIM criteria assessed from the nutritionDay survey [23], the frequency of dietary provision and clinical outcomes in relation to different nutritional statuses defined by the GLIM criteria are of concern in this study.

### 4.2. GLIM Diagnostic Flow Chart with Dietary Provision and Good 30-Day Outcome

Nutritional statuses of the 2010–2020 nutritionDay China cohort were systematically evaluated using the GLIM criteria. In the first stage of the malnutrition diagnostic scheme, we found that at least one of every three hospitalized patients was nutritionally at-risk, similar to previous studies in Brazil (32.8%) [31] and Vietnam (30.1%) [32], using the MUST as the risk screening tool. More than 20% of in-patients were defined as malnourished using the GLIM criteria, consistent with a cross-sectional study in elderly in-patients [33] and a reanalysis of a published prospective observational study [34]. However, the lowest frequency of good 30-day outcomes among the clusters of patients was observed in malnourished patients not receiving dietary provision, drawing attention to the association of dietary provision and malnutrition diagnosis with good 30-day outcomes.

### 4.3. Dietary Provision and Malnutrition Diagnosis Associated with Good 30-Day Outcome

The positive relationship between dietary provision and good 30-day outcomes was consistent in the univariate and multivariable analyses. In the multivariable models adjusting for other variables, patients receiving dietary provision had a nearly 1.5 times higher chance of obtaining a good 30-day outcome compared with those not receiving dietary provision. Dietary provision may, therefore, promote improved clinical outcomes. Moreover, compared with malnourished patients without dietary provision, malnourished and non-malnourished patients receiving dietary provision had a nearly 1.6 to 1.9 higher chance of achieving a good 30-day outcome, highlighting the potential impacts of dietary provision on in-patients. Notably, observational studies such as the nutritionDay surveys mainly show an association between dietary provision and good 30-day outcomes because some unmeasured factors, such as muscle mass and specific laboratory results [35,36,37], may have improved the prognosis of less severely ill patients who could receive dietary provision. Due to the potential prevention and treatment of dietary interventions on chronic diseases [11,12,13,14], hospital staff should take into account the impact of nutrition provision throughout medical care [38,39] on a good prognosis. This consideration could probably affect the nutritional choices of in-patients [40], who could be encouraged to eat meals with dietary provisions during hospitalization [41,42], especially among patients who are allowed to eat.

### 4.4. Cumulative Incidence of Good Outcome within 30 Days after nutritionDay

In terms of good outcomes, the non-malnourished patients had a significantly shortened median LOS after nutritionDay of 2 days compared with the malnourished patients; a similar trend was observed between the patients receiving and not receiving dietary provision. However, when combined with nutritional status and dietary provision, the malnourished patients receiving dietary provision had a significantly shortened median LOS after nutritionDay of 4 days compared to those not receiving dietary provision. Additionally, the non-malnourished patients receiving dietary provision had a median LOS after nutritionDay of 5 days, which was significantly shorter than the non-malnourished patients not receiving dietary provision. These findings reveal the importance of dietary provision during hospital stays. Tailored dietary provision needs to be delivered precisely while evaluating the nutritional status of in-patients to obtain better clinical outcomes [43]. Hospital staff should keep this in mind and carry it out flexibly as part of the nutrition management process for patients [44], even though they are malnourished with poor meal intake [45]. Specifically, the nutrition education framework should be created for patients, and the availability of a dietary provision practice platform for healthcare professionals should be increased in a benchmarking program designed for nutrition care [46,47].

### 4.5. Strengths and Limitations

The strengths of this study were that it validated the assessment of malnutrition according to the GLIM criteria using a large number of in-patients from the first decade of nutritionDay surveys in China and determined a relationship between dietary provision and clinical outcomes that can be compared to findings from other studies. However, several limitations in this study need to be noted. Firstly, observational data cannot determine a causal relationship between dietary provision and clinical outcome in a cross-sectional study. Secondly, the evaluations of nutritional status and intervention were based on self-reported data from a single-day cross-snapshot survey and are, therefore, prone to measurement errors due to a lack of periodic monitoring using objective measures, such as muscle mass and laboratory data. Thirdly, this study included participating hospitals that tend to be concerned about nutrition care, which might have introduced selection bias. Fourth, the LOS after nutritionDay in this study was calculated and analyzed instead of total LOS due to its length bias [6]. Fifth, we dichotomized the 30-day outcomes instead of one of the specific outcomes in the nutritionDay survey. Additionally, the 30-day clinical outcomes were limited. Further research on patients’ nutritional status would be worthwhile, including more body composition information and biochemical evaluations with long-term follow-up.

## 5. Conclusions

The results from nutritionDay surveys conducted in China from 2010 to 2020 provide evidence on dietary provision and GLIM-defined malnutrition associated with the 30-day clinical outcomes of in-patients. The results indicate a higher dietary provision in patients who consumed a full meal on nutritionDay. Importantly, dietary provision was associated with increased good 30-day outcomes compared to non-dietary provision, even in patients defined as malnourished according to the GLIM criteria. These results highlight the potential impacts of the dietary provision and nutritional status of in-patients on follow-up outcomes. Further nutrition care campaigns targeting specific dietary interventions are needed to translate this knowledge into action.

## Figures and Tables

**Table 1:** Demographic data of hospitalized patients, *n* = 5821.

	Median (IQR) or *n* (%)
Age, years, median (IQR)	58.0 (45.0–67.0)
Sex [female/male/unknown, *n* (%)]	2352 (40.4%)/3461 (59.5%)/8 (0.1%)
BMI, kg/m^2^, median (IQR)	22.8 (20.2–25.2)
BMI < 18.5 kg/m^2^, *n* (%)	710 (12.2%)
Surgical patients, *n* (%)	1797 (30.9%)
Weight loss within the last 3 months, *n* (%)	2246 (38.6%)
Less than half of normal food intake in the previous week, *n* (%)	1152 (19.8%)
Dietary provision [hospital food/special diet/both, *n* (%)]	3015 (51.8%)/ 2699 (46.4%)/239 (4.1%)/77 (1.3%)
Full meal eaten on nutritionDay, *n* (%)	2173 (37.3%)
Full meal not eaten on nutritionDay, *n* (%)	3372 (57.9%)
Full meal not eaten due to not being allowed to eat, *n* (%)	968 (16.6%)
Full meal not eaten due to decreased appetite, *n* (%)	930 (16.0%)
At risk of malnutrition defined by MUST, MUST ≥ 1, *n* (%)	1924 (33.1%)
Malnutrition defined by GLIM, *n* (%)	1328 (22.8%)
LOS after nutritionDay, days, median (IQR)	6.0 (3.0–12.0)
Good 30-day outcome, *n* (%)	5093 (87.5%)

IQR, interquartile range; BMI, body mass index; LOS, length of stay in hospital; MUST, Malnutrition Universal Screening Tool; GLIM, Global Leadership Initiative on Malnutrition.

**Table 2:** Nutritional provision and meal consumption on nutritionDay.

Eating on nutritionDay	*n*	Type of Nutritional Provision (Row Percentages)						
		Food/Diet			Multi-form of Food and Artificial Nutrition	No Food/Diet		Unsure/Missing
		Hospital food (regular and fortified/enriched hospital food)	Special diet	Multi-form of food and diet		Artificial nutrition ^a^	Nothing	
All	5821	46.4%	4.1%	1.3%	10.5%	23.2%	3.3%	11.2%
Eaten all	2173	64.5%	4.8%	1.7%	10.3%	8.2%	2.0%	8.4%
Eaten half	1176	56.5%	5.6%	1.9%	12.9%	12.3%	2.9%	7.8%
Eaten quarter	558	37.8%	6.1%	1.8%	17.2%	26.5%	2.0%	8.6%
Eaten nothing but allowed to eat	721	36.9%	3.6%	1.0%	8.7%	25.2%	4.7%	19.8%
Eaten nothing due to not being allowed to eat	917	9.9%	0.5%	0%	5.1%	63.8%	6.5%	14.1%
Missing data	276	23.2%	1.1%	0%	11.6%	40.6%	2.9%	20.7%

^a^ Artificial nutrition includes protein/energy supplements (e.g., ONS drinks), enteral nutrition, and parenteral nutrition.

**Table 3:** Cox regression models of dietary provision and malnutrition diagnosis associated with good 30-day outcomes.

		Model Ⅰ	Model II (Including GLIM)	Model III (Including Malnutrition Diagnosis with Dietary Provision)
Variable	Category	HR [95% CI]	HR [95% CI]	HR [95% CI]
Dietary provision	No food/diet	Reference	Reference	
	Food/diet	1.47 [1.35–1.60] ***	1.49 [1.38–1.61] ***	
	Multi-form of food and artificial nutrition	1.26 [1.13–1.41] ***	1.29 [1.16–1.43] ***	
	Unsure or missing	1.43 [1.28–1.60] ***	1.44 [1.29–1.61] ***	
Malnutrition defined by GLIM	No		Reference	
	Yes		0.83 [0.77–0.89] ***	
	Undefined		0.97 [0.88–1.08]	
Malnutrition diagnosis with dietary provision	Malnutrition without food/diet			Reference
	Malnutrition with food/diet			1.58 [1.36–1.83] ***
	Malnutrition with multi-form of food and artificial nutrition			1.34 [1.11–1.63] **
	Non-malnutrition without food/diet			1.29 [1.13–1.46] ***
	Non-malnutrition with food/diet			1.86 [1.64–2.11] ***
	Non-malnutrition with multi-form of food and artificial nutrition			1.56 [1.33–1.81] ***

Model I: Multivariable analysis with individual variables included in nutritionDay questionnaires. Model II: GLIM added to multivariable analysis without defined variables, including BMI, age, weight change within the last 3 months, major lesion types, food intake in the previous week, eaten on nutritionDay, and comorbidity. Model III: Malnutrition diagnosis with a dietary provision added to the multivariable analysis. All data are presented as HR and 95% CI. ** *p* < 0.01, *** *p* < 0.001. HR, hazard ratio; CI, confidence interval; LOS, length of stay in hospital; GLIM, Global Leadership Initiative on Malnutrition.

## Data Availability

The raw data supporting the conclusions of this article will be made available by the authors on request. The data are not publicly available as the authors are currently engaged in further exploration and analysis of these data, and hence have chosen not to disclose them publicly at this stage.

## Supplementary Materials

The following supporting information can be downloaded at: https://www.mdpi.com/article/10.3390/nu16040569/s1, Table S1: Nutritional status evaluation for nutritionDay 2010–2020 China survey; Table S2: Variables and median LOS after nutritionDay in patients between 30-day poor outcomes and good outcomes; Table S3: Cox regression analysis of 30-day good outcome; Table S4: Demographic characteristics in malnourished patients and non-malnourished patients.
