# Supplementary material for: Dietary Provision, GLIM-Defined Malnutrition and Their Association with Clinical Outcome: Results from the First Decade of nutritionDay in China
Source: Nutrients. 2024 Feb 19;16(4):569. doi: 10.3390/nu16040569 (PMC10893253; doi:10.3390/nu16040569)
Supplement: Supplementary file 1 [file nutrients-16-00569-s001.zip › Supplementary Table S1.pdf]

Supplementary Table S1. Nutritional status evaluation for nutritionDay 2010-2020 China survey

(a). MUST for nutritionDay 2010-2020 China survey [19]

| Score* | BMI ($kg/m^2$) | Weight loss in 3-6 months | Acute disease effect |
|---|---|---|---|
| 0 | > 20.0 | < 5% | Add a score of 2 if patient has acute disease and there has been or is likely to be no nutritional intake for > 5 days (i.e., about or less than a quarter to nearly nothing intake in the week before admission). |
| 1 | 18.5-20.0 | 5%-10% | |
| 2 | < 18.5 | > 10% | |
| *MUST ⩾ 1 is considered as at risk of malnutrition by adding scores of BMI, weight loss in 3-6 months and acute disease effect. | | | |

(b). GLIM criteria assess for nutritionDay 2010-2020 China survey [9,23]

| GLIM criteria assess for 2010-2020 nutritionDay | Phenotypic criteria | | Etiologic criteria | | |
|---|---|---|---|---|---|
| | Weight Loss (%) | BMI ($kg/m^2$) Asia | Food intake | | Inflammation (Acute disease/ injury or chronic disease-related) |
| Malnutrition (requires at least 1 phenotypic criterion &1 etiologic criterion when considered as at risk for malnutrition) | > 5% in 3 months<br>> 10% beyond 6 months | <18.5 if < 70y<br>< 20 if ⩾ 70y | ⩽ 75% intake for 1 (last) week | ⩽ 1/4 on nDay | Acute diseases: Emergency admissions AND ICD-10: 0100-Infectious parasitic diseases OR ICD-10 diagnosis: 0600-Nervous system OR 2000-External causes/accidents/assaults OR current infections OR Patients admitted to Trauma wards<br>Comorbidities: cancer OR cardiac insufficiency OR chronic lung disease OR chronic liver disease OR chronic kidney disease OR Other chronic disease. |

BMI, body mass index; GLIM, Global Leadership Initiative on Malnutrition; ICD, International Classification of Diseases; MUST, Malnutrition Universal Screening Tool.

References

**9.** Cederholm, T.; Jensen, G.L.; Correia, M.I.T.D.; Gonzalez, M.C.; Fukushima, R.; Higashiguchi, T.; Baptista, G.; Barazzoni, R.; Blaauw, R.; Coats, A.; et al. GLIM criteria for the diagnosis of malnutrition—A consensus report from the global clinical nutrition community. *Clin. Nutr.* **2019**, *38*, 1–9.

19. Stratton, R.J.; Hackston, A.; Longmore, D.; Dixon, R.; Price, S.; Stroud, M.; King, C.; Elia, M. Malnutrition in hospital outpatients and inpatients: Prevalence, concurrent validity and ease of use of the 'malnutrition universal screening tool' ('MUST') for adults. *Br. J. Nutr.* **2004**, *92*, 799–808.

23. Moick, S.; Hiesmayr, M.; Mouhieddine, M.; Kiss, N.; Bauer, P.; Sulz, I.; Singer, P.; Simon, J. Reducing the knowledge to action gap in hospital nutrition care—Developing and implementing NutritionDay 2.0. *Clin. Nutr.* **2021**, *40*, 936–945.
