# Supplementary material for: Dietary Provision, GLIM-Defined Malnutrition and Their Association with Clinical Outcome: Results from the First Decade of nutritionDay in China
Source: Nutrients. 2024 Feb 19;16(4):569. doi: 10.3390/nu16040569 (PMC10893253; doi:10.3390/nu16040569)
Supplement: Supplementary file 1 [file nutrients-16-00569-s001.zip › Supplementary Table S2.pdf]

Supplementary Table S2. Variables and median LOS after nutritionDay in patients between 30-day poor outcome and good outcome, *n* = 5821.

Variables	LOS after nutritionDay survey, days, median [Q1; Q3]	Poor outcome *n* (%)	Good outcome *n* (%)	*P* value
	6.0 [3.0; 12.0]	675	5093	
Hospital Location				< 0.001
Eastern China	6.0 [3.0; 12.0]	553 (81.9%)	3626 (71.2%)	
Southern China	6.0 [4.0; 11.0]	18 (2.7%)	88 (1.7%)	
Northern China	6.0 [4.0; 11.0]	97 (14.4%)	947 (18.6%)	
Northwestern China	4.0 [1.0; 7.0]	7 (1.0%)	432 (8.5%)	
Department				< 0.001
General surgery	6.0 [3.0; 12.0]	352 (52.1%)	2273 (44.6%)	
Orthopedics	8.0 [4.0; 13.0]	18 (2.7%)	127 (2.5%)	
Urological surgical	5.0 [2.0; 8.0]	1 (0.1%)	75 (1.5%)	
Neurosurgery	9.0 [5.0; 18.0]	50 (7.4%)	272 (5.3%)	
Gastroenterology and hepatology	5.0 [2.0; 9.0]	62 (9.2%)	920 (18.1%)	
Respiratory Medicine	6.0 [3.0; 11.0]	6 (0.9%)	60 (1.2%)	
Oncology	6.0 [3.0; 11.0]	95 (14.1%)	605 (11.9%)	
Neurology	6.0 [4.0; 12.0]	15 (2.2%)	180 (3.5%)	
Geriatrics	11.0 [6.0; 19.0]	15 (2.2%)	48 (0.9%)	
Cardiothoracic surgery	8.0 [4.0; 13.0]	15 (2.2%)	175 (3.4%)	
Nephrology	5.0 [1.0; 9.0]	24 (3.6%)	236 (4.6%)	
Others [a]	7.0 [4.0; 13.0]	22 (3.3%)	122 (2.4%)	
Sex				0.070
Female	6.0 [3.0; 12.0]	251 (37.2%)	2079 (40.8%)	
Male	6.0 [3.0; 12.0]	422 (62.5%)	3008 (59.1%)	
Missing	4.0 [2.5; 7.5]	2 (0.3%)	6 (0.1%)	
Age				< 0.01
18-29 years	5.0 [2.0; 12.0]	62 (9.2%)	385 (7.6%)	
30-39 years	6.0 [3.0; 12.0]	90 (13.3%)	488 (9.6%)	
40-49 years	6.0 [3.0; 11.0]	92 (13.6%)	747 (14.7%)	
50-59 years	6.0 [3.0; 12.0]	124 (18.4%)	1139 (22.4%)	
60-69 years	6.0 [3.0; 11.0]	162 (24.0%)	1319 (25.9%)	
70-79 years	6.0 [3.0; 12.0]	101 (15.0%)	745 (14.6%)	
⩾ 80 years	7.0 [4.0; 12.0]	44 (6.5%)	270 (5.3%)	
BMI				< 0.001
18.5-24.9	6.0 [3.0; 12.0]	382 (56.6%)	3066 (60.2%)	
< 18.5	7.0 [3.0; 13.0]	146 (21.6%)	560 (11.0%)	
25.0-29.9	5.0 [2.0; 11.0]	124 (18.4%)	1237 (24.3%)	

⩾ 30.0	5.0 [3.0; 11.0]	17 (2.5%)	164 (3.2%)	
Missing	5.0 [2.0; 8.0]	6 (0.9%)	66 (1.3%)	
LOS before nutritionDay				< 0.001
0-6 days	6.0 [3.0; 11.0]	233 (34.5%)	2577 (50.6%)	
7-13 days	6.0 [2.0; 11.0]	159 (23.6%)	1233 (24.2%)	
14-20 days	6.0 [3.0; 12.5]	78 (11.6%)	471 (9.2%)	
⩾ 21 days	8.0 [4.0; 17.0]	163 (24.1%)	403 (7.9%)	
Missing	5.0 [3.0; 9.0]	42 (6.2%)	409 (8.0%)	
Ever stayed in ICU				< 0.001
No	6.0 [3.0; 11.0]	556 (82.4%)	4279 (84.0%)	
Yes	6.0 [3.0; 13.0]	116 (17.2%)	695 (13.6%)	
Missing	8.0 [4.0; 13.0]	3 (0.4%)	119 (2.3%)	
End-stage disease				0.104
No	6.0 [3.0; 12.0]	578 (85.6%)	4337 (85.2%)	
Yes	5.0 [2.0; 11.0]	40 (5.9%)	231 (4.5%)	
Unknown or missing	6.0 [2.3; 12.0]	57 (8.4%)	525 (10.3%)	
Mobility				< 0.001
Walk without assistance	6.0 [2.0; 11.0]	407 (60.3%)	3800 (74.6%)	
Walk with assistance	7.0 [4.0; 13.0]	132 (19.6%)	689 (13.5%)	
Bedridden	7.0 [4.0; 13.0]	113 (16.7%)	333 (6.5%)	
Missing	6.0 [3.0; 12.0]	23 (3.4%)	271(5.3%)	
Self-rated health				< 0.001
Excellent and very good	5.0 [2.5; 11.0]	42 (6.2%)	400 (7.9%)	
Good	5.0 [2.0; 11.0]	156 (23.1%)	1677 (32.9%)	
Fair	6.0 [3.0; 12.0]	358 (53.0%)	2337 (45.9%)	
Poor and very poor	7.0 [4.0; 13.0]	111 (16.4%)	603 (11.8%)	
Missing	8.0 [4.0; 16.0]	8 (1.2%)	76 (1.5%)	
Food intake in the previous week				< 0.001
More than normal or normal	6.0 [3.0; 11.0]	369 (54.7%)	3430 (67.3%)	
A little less than normal	6.0 [3.0; 12.0]	81 (12.0%)	641 (12.6%)	
Less than half of normal	6.0 [3.0; 13.0]	215 (31.9%)	926 (18.2%)	
Missing	7.0 [4.0; 14.0]	10 (1.5%)	96 (1.9%)	
Eating on nutritionDay				< 0.001
About all	6.0 [3.0; 12.0]	183 (27.1%)	1977 (38.8%)	
1/2	5.0 [2.0; 10.0]	110 (16.3%)	1061 (20.8%)	
1/4	5.0 [2.0; 10.0]	96 (14.2%)	456 (9.0%)	

Nothing	6.0 [4.0; 12.0]	254 (37.6%)	1367 (26.8%)	
Missing	7.0 [4.0; 14.0]	32 (4.7%)	232 (4.6%)	
Number of drugs before admission				< 0.001
None	5.0 [2.0; 11.0]	165 (24.4%)	1565 (30.7%)	
1-2	6.0 [3.0; 12.0]	211 (31.3%)	1611 (31.6%)	
3-5	6.0 [3.0; 11.0]	125 (18.5%)	1046 (20.5%)	
＞5	6.0 [4.0; 13.0]	109 (16.1%)	531 (10.4%)	
Unknown or missing	6.5[3.0; 13.0]	65 (9.6%)	340 (6.7%)	
Major lesion types				< 0.001
Cancer	6.0 [3.0; 12.0]	190 (28.1%)	1266 (24.9%)	
Neurological disease	8.0 [4.0; 14.0]	51 (7.6%)	339 (6.7%)	
Digestive disease	5.0 [2.0; 11.0]	258 (38.2%)	1971 (38.7%)	
Endocrine/nutritional/metabolic disease	5.0 [4.0; 14.0]	15 (2.2%)	100 (2.0%)	
Cardiovascular disease	7.0 [3.0; 13.0]	25 (3.7%)	161 (3.2%)	
Respiratory disease	6.0 [3.0; 10.3]	16 (2.4%)	130 (2.6%)	
Genitourinary disease	5.0 [2.0; 11.0]	27 (4.0%)	242 (4.8%)	
Other	6.0 [3.0; 13.0]	74 (11.0%)	475 (9.3%)	
Missing	5.0 [2.0; 10.0]	19 (2.8%)	409 (8.0%)	
Comorbidity				< 0.001
No	5.0 [2.0; 11.0]	186 (27.6%)	1703 (33.4%)	
Yes	6.0 [4.0; 12.0]	466 (69.0%)	2895 (56.8%)	
Missing	5.0 [2.0; 9.3]	23 (3.4%)	495 (9.7%)	
Surgical status				0.299
Non-surgical	6.0 [3.0; 11.0]	338 (50.1%)	2350 (46.1%)	
Preoperative	7.0 [4.0; 14.0]	139 (20.6%)	1135 (22.3%)	
Postoperative	5.0 [2.0; 10.0]	195 (28.9%)	1578 (31.0%)	
Undefined or missing	5.0 [4.0; 11.0]	3 (0.4%)	30 (0.6%)	
Dietary provision				< 0.001
No food/diet	7.0 [4.0; 14.0]	292 (43.3%)	1236 (24.3%)	
Food/diet	5.0 [2.0; 11.0]	253 (37.5%)	2748 (54.0%)	
Multi-form of food and artificial nutrition	6.0 [3.0; 11.0]	57 (8.4%)	542 (10.6%)	
Unsure or missing	6.0 [3.0; 11.0]	73 (10.8%)	567 (11.1%)	
Weight change within last 3 months				< 0.001
Stable weight	5.0 [2.0; 11.0]	244 (36.1%)	2343 (46.0%)	
Weight loss	6.0 [3.0; 12.0]	326 (48.3%)	1906 (37.4%)	

Weight gain	5.0 [2.0; 9.0]	39 (5.8%)	300 (5.9%)	
Unsure or missing	7.0 [4.0; 14.0]	66 (9.8%)	544 (10.7%)	
At risk of malnutrition defined by MUST				< 0.001
No	5.5 [3.0; 11.0]	312 (46.2%)	3023 (59.4%)	
Yes	6.0 [3.0; 13.0]	320 (47.4%)	1590 (31.2%)	
Undefined	6.0 [3.0; 13.0]	43 (6.4%)	480 (9.4%)	
Malnutrition defined by GLIM				< 0.001
No	6.0 [3.0; 11.0]	375 (55.6%)	3552 (69.7%)	
Yes	7.0 [4.0; 13.0]	257 (38.1%)	1061 (20.8%)	
Undefined	6.0 [3.0; 13.0]	43 (6.4%)	480 (9.4%)	

[a] Others includes endocrinology, emergency, burn surgery, otolaryngology, internal medicine, stomatology and trauma departments which with fewer than 60 patients.

Values were presented as Median [Q1; Q3] or *n* (%); *P* value, poor outcome group vs. good outcome group. Missing data were excluded. LOS, length of stay in hospital; BMI, body mass index; ICU, intensive care unit; MUST, Malnutrition Universal Screening Tool; GLIM, Global Leadership Initiative on Malnutrition.
