# Supplementary material for: Dietary Provision, GLIM-Defined Malnutrition and Their Association with Clinical Outcome: Results from the First Decade of nutritionDay in China
Source: Nutrients. 2024 Feb 19;16(4):569. doi: 10.3390/nu16040569 (PMC10893253; doi:10.3390/nu16040569)
Supplement: Supplementary file 1 [file nutrients-16-00569-s001.zip › Supplementary Table S3.pdf]

Supplementary Table S3. Cox regression analysis of 30-day good outcome.

Variable	Category	Univariate analysis	Model Ⅰ	Model Ⅱ (Including GLIM)	Model Ⅲ (Including malnutrition diagnosis with nutrition intake)
		HR [95% CI]	HR [95% CI]	HR [95% CI]	HR [95% CI]
**Sex**	Female	Reference	Reference	Reference	Reference
	Male	0.94 [0.89-1.00]*	0.94 [0.88-0.99]*	0.94 [0.89-1.00]*	0.94 [0.89-1.01]
	Missing	0.78 [0.35-1.73]	0.54 [0.24-1.21]	0.61 [0.27-1.37]	1.35 [0.55-3.28]
**Age**	18-29 years	Reference			
	30-39 years	0.96 [0.84-1.10]			
	40-49 years	1.12 [0.99-1.27]			
	50-59 years	1.11 [0.99-1.24]			
	60-69 years	1.08 [0.96-1.21]			
	70-79 years	1.03 [0.91-1.17]			
	⩾ 80 years	0.90 [0.77-1.05]			
**BMI, kg/m²**	18.5-24.9	Reference	Reference		
	< 18.5	0.75 [0.69-0.82]***	0.86 [0.78-0.95]**		
	25.0-29.9	1.13 [1.05-1.20]***	1.10 [1.03-1.18]**		
	⩾ 30.0	1.15 [0.98-1.35]	1.10 [0.94-1.30]		
	Missing	1.30 [1.02-1.66]*	1.19 [0.92-1.53]		
**Major lesion types**	Cancer	1.11 [1.00-1.24]	0.88 [0.77-1.00]		
	Neurological disease	0.97 [0.85-1.12]	0.94 [0.77-1.14]		
	Digestive disease	1.22 [1.10-1.35]***	0.95 [0.84-1.07]		

	Endocrine/ nutritional/ metabolic disease	1.05 [0.84-1.30]	0.75 [0.59-0.96]*		
	Cardiovascular disease	0.98 [0.82-1.18]	1.00 [0.82-1.23]		
	Respiratory disease	1.17 [0.96-1.42]	1.00 [0.78-1.29]		
	Genitourinary disease	1.28 [1.10-1.50]**	0.66 [0.49-0.87]**		
	Other	Reference	Reference		
	Missing	1.56 [1.36-1.79]***	1.55 [1.23-1.95]***		
**Comorbidity**	No	Reference	Reference		
	Yes	0.84 [0.79-0.89]***	0.85 [0.79-0.91]***		
	Missing	1.25 [1.13-1.38]***	1.05 [0.88-1.26]		
**Previous ICU stay**	No	Reference	Reference	Reference	Reference
	Yes	0.86 [0.79-0.93]***	1.00 [0.91-1.10]	1.03 [0.94-1.13]	1.01 [0.91-1.12]
	Missing	1.03 [0.84-1.27]	1.02 [0.83-1.27]	1.01 [0.82-1.25]	1.10 [0.84-1.44]
**Mobility**	Walk without assistance	Reference	Reference	Reference	Reference
	Walk with assistance	0.78 [0.71-0.84]***	0.90 [0.82-0.98]*	0.90 [0.83-0.98]*	0.90 [0.82-0.99]*
	Bedridden	0.59 [0.53-0.66]***	0.75 [0.66-0.85]***	0.74 [0.65-0.83]***	0.72 [0.62-0.83]***
	Missing	0.93 [0.82-1.06]	1.01 [0.87-1.18]	1.01 [0.87-1.17]	1.07 [0.91-1.27]
**Self-rated health**	Excellent and very good	0.92 [0.82-1.03]	0.91 [0.82-1.02]	0.92 [0.82-1.03]	0.96 [0.85-1.09]
	Good	Reference	Reference	Reference	Reference
	Fair	0.81 [0.76-0.87]***	0.91 [0.85-0.97]**	0.89 [0.83-0.95]***	0.89 [0.83-0.96]**
	Poor and very poor	0.73 [0.67-0.80]***	0.98 [0.88-1.09]	0.92 [0.83-1.02]	0.94 [0.84-1.05]

	Missing	0.69 [0.53-0.89]**	0.81 [0.59-1.12]	0.78 [0.59-1.03]	0.80 [0.54-1.16]
**Surgical status**	Non-surgical	Reference	Reference	Reference	Reference
	Preoperative	0.90 [0.83-0.96]**	0.82 [0.75-0.89]***	0.82 [0.76-0.89]***	0.79 [0.72-0.87]***
	Postoperative	1.18 [1.10-1.26]***	1.34 [1.24-1.45]***	1.31 [1.21-1.41]***	1.33 [1.22-1.45]***
	Undefined or missing	1.21 [0.74-1.99]	0.94 [0.57-1.57]	0.99 [0.60-1.63]	0.81 [0.45-1.45]
**LOS before nutritionDay**	0-6 days	Reference	Reference	Reference	Reference
	7-13 days	0.96 [0.90-1.03]	0.99 [0.92-1.07]	0.99 [0.92-1.07]	0.98 [0.91-1.07]
	14-20 days	0.87 [0.79-0.96]**	0.95 [0.85-1.06]	0.94 [0.85-1.05]	0.97 [0.86-1.09]
	⩾ 21 days	0.51 [0.46-0.57]***	0.62 [0.55-0.69]***	0.62 [0.55-0.69]***	0.62 [0.55-0.71]***
	Missing	1.03 [0.93-1.15]	0.81 [0.68-0.96]*	0.82 [0.69-0.97]*	0.86 [0.72-1.02]
**Number of drugs before admission**	None	Reference	Reference	Reference	Reference
	1-2	0.93 [0.87-1.00]*	1.03 [0.95-1.11]	1.00 [0.93-1.07]	1.03 [0.95-1.11]
	3-5	0.95 [0.88-1.03]	1.08 [0.99-1.17]	1.05 [0.96-1.14]	1.09 [1.00-1.20]
	> 5	0.75 [0.68-0.83]***	1.01 [0.90-1.13]	0.95 [0.85-1.06]	0.96 [0.85-1.08]
	Unknown or missing	0.76 [0.67-0.86]***	1.02 [0.89-1.17]	0.95 [0.83-1.08]	0.97 [0.83-1.13]
**Weight change within last 3 months**	Stable weight	Reference	Reference		
	Weight loss	0.80 [0.75-0.85]***	0.94 [0.87-1.00]		
	Weight gain	0.99 [0.88-1.12]	1.02 [0.90-1.16]		
	Unsure or missing	0.80 [0.73-0.88]***	0.87 [0.78-0.96]**		

**Food intake in the previous week**	More than normal or normal	Reference	Reference		
	A little less than normal	0.89 [0.82-0.97]**	0.94 [0.86-1.03]		
	Less than half of normal	0.75 [0.70-0.81]***	0.87 [0.80-0.95]**		
	Missing	0.85 [0.68-1.06]	1.03 [0.78-1.36]		
**Eating on nutritionDay**	About all	Reference	Reference		
	1/2	1.04 [0.97-1.12]	1.09 [1.00-1.18]*		
	1/4	0.92 [0.83-1.02]	1.05 [0.94-1.18]		
	Nothing	0.82 [0.76-0.87]***	0.94 [0.86-1.02]		
	Missing	0.75 [0.65-0.86]***	0.99 [0.83-1.18]		
**Dietary provision**	No food/diet	Reference	Reference	Reference	
	Food/diet	1.55 [1.45-1.66]***	1.47 [1.35-1.60]***	1.49 [1.38-1.61]***	
	Multi-form of food and artificial nutrition	1.39 [1.26-1.54]***	1.26 [1.13-1.41]***	1.29 [1.16-1.43]***	
	Unsure or missing	1.41 [1.28-1.57]***	1.43 [1.28-1.60]***	1.44 [1.29-1.61]***	
**Malnutrition defined by GLIM**	No			Reference	
	Yes			0.83 [0.77-0.89]***	
	Undefined			0.97 [0.88-1.08]	
**Malnutrition diagnosis with dietary provision**	Malnutrition without food/diet				Reference
	Malnutrition with food/diet				1.58 [1.36-1.83]***

Malnutrition with multi-form of food and nutritional support	1.34 [1.11-1.63]**
Non-malnutrition without food/diet	1.29 [1.13-1.46]***
Non-malnutrition with food/diet	1.86 [1.64-2.11]***
Non-malnutrition with multi-form of food and nutritional support	1.56 [1.33-1.81]***

Model Ⅰ: Multivariable analysis with individual variables included in nutritionDay questionnaires. Model Ⅱ: GLIM added to multivariable analysis without defined variables including of BMI, age, weight change within last 3 months, major lesion types, food intake in the previous week, eaten on nutritionDay and comorbidity. Model Ⅲ: Malnutrition diagnosis defined by GLIM with dietary provision added to multivariable analysis. All data are presented as HR and 95% CI. * $p < 0.05$, ** $p < 0.01$, *** $p < 0.001$. HR, hazard ratio; CI, confidence interval; BMI, body mass index; LOS, length of stay in hospital; GLIM, Global Leadership Initiative on Malnutrition.
