# Supplementary material for: Dietary Provision, GLIM-Defined Malnutrition and Their Association with Clinical Outcome: Results from the First Decade of nutritionDay in China
Source: Nutrients. 2024 Feb 19;16(4):569. doi: 10.3390/nu16040569 (PMC10893253; doi:10.3390/nu16040569)
Supplement: Supplementary file 1 [file nutrients-16-00569-s001.zip › Supplementary Table S4.pdf]

Supplementary Table S4. Demographic characteristics in malnourished patients and non-malnourished patients, n = 5821.

Variables	All patients<br>n (%)	Non-malnourished patients<br>n (%)	Malnourished patients<br>n (%)	P value
	5821	3962	1328	
Sex				0.35
Female	2352 (40.4%)	1590 (40.1%)	564 (42.5%)	
Male	3461(59.5%)	2366 (59.7%)	762 (57.4%)	
Missing	8 (0.1%)	6 (0.2%)	2 (0.2%)	
Age				< 0.001
18-29 years	451 (7.7%)	282 (7.1%)	148 (11.1%)	
30-39 years	583 (10.0%)	415 (10.5%)	127 (9.6%)	
40-49 years	844 (14.5%)	608 (15.3%)	169 (12.7%)	
50-59 years	1269 (21.8%)	924 (23.3%)	233 (17.5%)	
60-69 years	1505 (25.9%)	1050 (26.5%)	318 (23.9%)	
70-79 years	850 (14.6%)	516 (13.0%)	226 (17.0%)	
⩾ 80 years	319 (5.5%)	167 (4.2%)	107 (8.1%)	
BMI				0.009
18.5-24.9	3472 (59.6%)	2554 (64.5%)	611 (46.0%)	
< 18.5	710 (12.2%)	121 (3.1%)	589 (44.4%)	
25.0-29.9	1381 (23.7%)	1128 (28.5%)	115 (8.7%)	
⩾ 30.0	184 (3.2%)	159 (4.0%)	12 (0.9%)	
Missing	74 (1.3%)	0 (0.0%)	1 (0.1%)	
Surgical status				< 0.001
Non-surgical	2704 (46.5%)	1717 (43.3%)	715 (53.8%)	
Preoperative	1286 (22.1%)	953 (24.1%)	217 (16.3%)	
Postoperative	1797 (30.9%)	1273 (32.1%)	389 (29.3%)	
Undefined or missing	34 (0.6%)	19 (0.5%)	7 (0.5%)	
Weight change within last 3 months				< 0.001
Stable weight	2607 (44.8%)	2356 (59.5%)	218 (16.4%)	
Weight loss	2246 (38.6%)	1129 (28.5%)	1010 (76.1%)	
Weight gain	340 (5.8%)	310 (7.8%)	29 (2.2%)	
Unsure or missing	628 (10.8%)	167 (4.2%)	71 (5.4%)	
Dietary provision				< 0.001

No food/diet	1541 (26.5%)	871 (22.0%)	554 (41.7%)	
Food/diet	3015 (51.8%)	2249 (56.8%)	455 (34.3%)	
Multi-form of food and artificial nutrition	613 (10.5%)	388 (9.8%)	185 (13.9%)	
Unsure or missing	652 (11.2%)	454 (11.5%)	134 (10.1%)	
Food intake in the previous week				< 0.001
More than normal or normal	3825 (65.7%)	2968 (74.9%)	513 (38.6%)	
A little less than normal	726 (12.5%)	435 (11.0%)	241 (18.1%)	
Less than half of normal	1152 (19.8%)	506 (12.8%)	554 (41.7%)	
Missing	118 (2.0%)	53 (1.3%)	20 (1.5%)	
Eating on nutritionDay	276 (4.7%)	153 (3.9%)	62 (4.7%)	< 0.001
About all	2173 (37.3%)	1760 (44.4%)	224 (16.9%)	
1/2	1176 (20.2%)	833 (21.0%)	235 (17.7%)	
1/4	558 (9.6%)	294 (7.4%)	216 (16.3%)	
Nothing	1638 (28.1%)	922 (23.3%)	591 (44.5%)	
Missing	276 (4.7%)	153 (3.9%)	62 (4.7%)	
30-day outcomes				< 0.001
Poor outcome	675 (11.6%)	375 (9.5%)	257 (19.4%)	
Good outcome	5093 (87.5%)	3552 (89.7%)	1061 (79.9%)	
Missing	53 (0.9%)	35 (0.9%)	10 (0.8%)	

P value, non-malnourished patients vs. malnourished patients. Missing data were excluded. BMI, body mass index
